# A methodology for identifying high‐need, high‐cost patient personas for international comparisons

**DOI:** 10.1111/1475-6773.13890

**Published:** 2021-11-10

**Authors:** Jose F. Figueroa, Kathryn E. Horneffer, Kristen Riley, Olukorede Abiona, Mina Arvin, Femke Atsma, Enrique Bernal‐Delgado, Carl Rudolf Blankart, Nicholas Bowden, Sarah Deeny, Francisco Estupiñán‐Romero, Robin Gauld, Tonya Moen Hansen, Philip Haywood, Nils Janlov, Hannah Knight, Luca Lorenzoni, Alberto Marino, Zeynep Or, Leila Pellet, Duncan Orlander, Anne Penneau, Andrew J. Schoenfeld, Kosta Shatrov, Kjersti Eeg Skudal, Mai Stafford, Onno van de Galien, Kees van Gool, Walter P. Wodchis, Marit Tanke, Ashish K. Jha, Irene Papanicolas

**Affiliations:** ^1^ Department of Health Policy and Management Harvard T.H. Chan School of Public Health Boston Massachusetts USA; ^2^ Centre for Health Economics Research and Evaluation (CHERE) University of Technology Sydney Australia; ^3^ Radboud University Medical Center, Radboud Institute for Health Sciences, Scientific Center for Quality of Healthcare Nijmegen The Netherlands; ^4^ Institute for Health Sciences in Aragon (IACS) Zaragoza Spain; ^5^ KPM Center for Public Management University of Bern Bern Switzerland; ^6^ Hamburg Center for Health Economics Universität Hamburg Hamburg Germany; ^7^ Dunedin School of Medicine University of Otago Dunedin New Zealand; ^8^ The Health Foundation London UK; ^9^ Otago Business School University of Otago Dunedin New Zealand; ^10^ Norwegian Institute of Public Health Oslo Norway; ^11^ The Swedish Agency for Health and Care Services Analysis Stockholm Sweden; ^12^ Health Division Organisation for Economic Co‐operation and Development (OECD) Paris France; ^13^ Department of Health Policy London School of Economics London UK; ^14^ Institute for Research and Documentation in Health Economics (IRDES) Paris France; ^15^ Department of Orthopedic Surgery Brigham and Women's Hospital Boston Massachusetts USA; ^16^ Zilveren Kruis Leusden The Netherlands; ^17^ Institute of Health Policy Management & Evaluation University of Toronto Toronto Ontario Canada; ^18^ Institute for Better Health, Trillium Health Partners Mississauga Ontario Canada; ^19^ Brown School of Public Health Providence Rhode Island USA

**Keywords:** international comparison, vignettes

## Abstract

**Objective:**

To establish a methodological approach to compare two high‐need, high‐cost (HNHC) patient personas internationally.

**Data sources:**

Linked individual‐level administrative data from the inpatient and outpatient sectors compiled by the International Collaborative on Costs, Outcomes, and Needs in Care (ICCONIC) across 11 countries: Australia, Canada, England, France, Germany, the Netherlands, New Zealand, Spain, Sweden, Switzerland, and the United States.

**Study design:**

We outline a methodological approach to identify HNHC patient types for international comparisons that reflect complex, priority populations defined by the National Academy of Medicine. We define two patient profiles using accessible patient‐level datasets linked across different domains of care—hospital care, primary care, outpatient specialty care, post‐acute rehabilitative care, long‐term care, home‐health care, and outpatient drugs. The personas include a frail older adult with a hip fracture with subsequent hip replacement and an older person with complex multimorbidity, including heart failure and diabetes. We demonstrate their comparability by examining the characteristics and clinical diagnoses captured across countries.

**Data collection/extraction methods:**

Data collected by ICCONIC partners.

**Principal findings:**

Across 11 countries, the identification of HNHC patient personas was feasible to examine variations in healthcare utilization, spending, and patient outcomes. The ability of countries to examine linked, individual‐level data varied, with the Netherlands, Canada, and Germany able to comprehensively examine care across all seven domains, whereas other countries such as England, Switzerland, and New Zealand were more limited. All countries were able to identify a hip fracture persona and a heart failure persona. Patient characteristics were reassuringly similar across countries.

**Conclusion:**

Although there are cross‐country differences in the availability and structure of data sources, countries had the ability to effectively identify comparable HNHC personas for international study. This work serves as the methodological paper for six accompanying papers examining differences in spending, utilization, and outcomes for these personas across countries.

**What is known on this topic**

- International comparisons of health systems mostly rely on comparisons of the inpatient setting.
- Little comparative work examines patterns of spending and utilization of high‐need, high‐cost (HNHC) patients across different components of the healthcare system, despite constituting a priority group for policymakers.
- Vignette methodologies are a useful way to compare resource use for similar types of patients across countries.

**What this study adds**

- This study presents a framework and methodology for examining differences in spending, utilization and patient outcomes for specific types of priority high‐need, high‐cost patients across countries
- This study serves as the methodological paper for an international comparison series of six other papers that examines differences across different care settings, including hospital care, primary care, outpatient specialty care, post‐acute rehabilitative care, long‐term care, home‐health care, and outpatient drugs.
- Although there are cross‐country differences in the availability and structure of data sources, countries had the ability to effectively identify comparable HNHC personas for international study.

## 1 INTRODUCTION

International comparisons of patient trajectories across health systems can be a useful tool to help national policymakers understand whether countries are achieving comparable outcomes at similar costs for their populations. To date, most international efforts have largely focused on understanding variation across individual conditions or episodes of care in the inpatient setting for general populations.^1^, ^2^, ^3^, ^4^, ^5^, ^6^ Other work focused on evaluating end‐of‐life care in people with cancer and revealed considerable variation in the use of intensive and hospital‐centric care across high‐income countries.

However, the lack of available and similarly structured patient‐level information for specific types of high‐need patients across the entire care trajectory limits the potential to identify improvements to be made across the health system. For these patients in particular, it is important for policymakers to understand how care is distributed across settings, such as primary care, outpatient specialty care, and even long‐term care, and how use in one setting may influence utilization in another. Understanding which health systems are more effective at managing specific types of HNHC populations could offer key insights to address rising costs, waste, and inequities in the system, as well as improve patient outcomes.

In order to address this challenge, the International Collaborative on Costs, Outcomes and Needs in Care (ICCONIC) was formed in 2018. In this article, we put forward a methodological framework to enable the cross‐country comparison of resource use and outcomes for specific types of HNHC patients across the entire patient pathway. Our methodology builds upon previous international comparisons work and utilizes a clinical vignette approach,^3^, ^4^, ^6^, ^7^ which allows for the systematic collection and comparison of data across countries with different structures of patient‐level datasets.

Specifically, we had three key objectives. First, we outline an approach for selecting two types of HNHC patient “personas,” drawing on a typology put forward by the National Academy of Medicine (NAM), to be used as tracers across different countries and health systems.^8^ Second, we propose a detailed clinical vignette to be used across countries to identify two types of HNHC personas using available and accessible patient‐level datasets that allow for the comparison of utilization, spending, and patient outcomes across countries. Finally, we demonstrate the comparability of these two specific personas—(1) an older adult with frailty who sustains a hip fracture and subsequent hip replacement or osteosynthesis, and (2) an older person with complex multimorbidity, specifically a person hospitalized with heart failure and a comorbidity of diabetes—across the 11 countries in the ICCONIC collaborative. Importantly, this work provides the methodological framework used in an accompanying six original research manuscripts copublished in the *Health Services Research* “Special Issue on International Comparisons of High Need, High‐Cost Patients.”^9^, ^10^, ^11^, ^12^, ^13^, ^14^ These six original research articles examine detailed variation in spending, utilization, and patient outcomes of the two specific high‐need patient cohorts across different care settings.

## 2 METHODOLOGY AND APPROACH

### 2.1 Formation of the ICCONIC collaborative

To carry out this work, we formed the ICCONIC research collaborative in 2018 where we brought together partners from each of the 11 countries, representing a wide range of institutions, including universities, healthcare providers, think tanks, research centers, and international organizations.^15^ The research partners included collaborators with experience using routine data to compare healthcare performance at the international level and access to the datasets of interest for the study of HNHC patients.^16^, ^17^, ^18^, ^19^, ^20^ The 11 participating countries—Australia, Canada, England, France, Germany, the Netherlands, New Zealand, Spain, Sweden, Switzerland, and the United States—all represent high‐income countries with high expenditures on health care, but also healthcare systems that are funded and organized differently. For a list of important health system differences, please see Table A1.

Our methodological approach to examine variations in resource use for HNHC personas combines two existing approaches that are relatively novel for international comparison of health systems. First, we propose to use linked, patient‐level data to examine the entire care pathway, rather than focusing only on care in the hospital setting allowing us to trace the resources used by patients across the system. Second, our unit of analysis for comparison is the patient, who we follow throughout the system. This approach builds on the use of clinical vignette methodologies to identify similar cohorts of patients, as have been used by other projects to examine resource use in the inpatient setting,^3^, ^4^, ^5^, ^7^ and by international organizations to examine variations in clinical practice.^21^, ^22^

To advise the conceptual and methodological approach, and comment on the results, we formed an advisory board consisting of national and international experts within each of the 11 countries. The members include health economists, health services researchers, clinicians, policymakers, and representatives from payers of healthcare services (see Table A2).

### 2.2 Defining the HNHC patient personas

The first step of the project was to identify a group of HNHC patient subtypes to trace through the different health systems using a predefined clinical vignette, which in this article, we refer to as “HNHC patient personas.” This step is necessary for two reasons. First, HNHC patients are not a homogenous group, and while they include patients with substantial clinical need, their care needs will differ. In order to identify more actionable insights for policymakers and practitioners, we wanted to focus on certain types of HNHC patients that were defined by the same types of need. The second reason we focus on distinct HNHC patients is to ensure comparability of the patient cohorts across countries. This is because the composition of HNHC patient types may vary across countries. Therefore, looking at care trajectories and outcomes of this broader group may produce misleading policy recommendations.

To identify these HNHC patient personas, we defined clinical vignettes that were based from the NAM typology of HNHC priority populations.^8^ The NAM recently identified priority groups of patients that were among the most expensive to care for have substantial healthcare needs, and are particularly vulnerable to poor‐quality care.^23^, ^24^, ^25^ Based on the NAM framework, we selected two specific HNHC patient profiles that we believed would be most identifiable across countries, given existing data collection and coding systems, and identified specific types of patients that would belong to this category using a clinical vignette approach. The first included an older adult with frailty (defined by the following clinical vignette: person above age 65 who is hospitalized with hip fracture and received a subsequent hip replacement), and a person with major complex comorbidities (defined by the following clinical vignette: a person between the ages of 65 and 90 hospitalized with heart failure and a comorbidity of diabetes) (Table 1). Both of these clinical vignettes were identifiable through an inpatient admission, which are more consistently coded through more comparable coding systems (mostly deriving from the WHO ICD‐10 code system) than those in other settings (e.g., primary care, outpatient care). These decisions were made through a consensus decision‐making process by all members in the collaborative, which included physicians, policymakers, data scientists, statisticians, and health economists.

**TABLE 1:** Identification of high‐need, high‐cost patient personas for international comparison

National Academy of Medicine Priority Population	Identified high‐need patient personas for comparison	Age group	Identification with diagnostic codes
Frail older person	Older person with hip fracture	65 years and above	Primary diagnoses of hospitalization S72.0: Fracture of neck of femur S72.1: Pertrochanteric fracture S72.2: Subtrochanteric fracture Procedures (using country‐specific codes) Total hip replacement Partial hip replacement Osteosynthesis/pinning
Person with complex multimorbidity	Older person admitted with a heart failure exacerbation with a comorbidity of diabetes	65–90 years	Primary diagnosis of hospitalization I50: Heart failure Secondary diagnosis of diabetes E11‐E14

*Note*: Across most countries, the diagnostic classification system of ICD‐10‐WHO codes was used. Spain used ICD‐9 codes, whereas the Netherlands used a customized approach to identify relevant codes using input from clinical experts in private insurer data.

### 2.3 Identifying HNHC personas across countries' datasets

In order to identify and follow each of these personas across their pathway of care over a period of a year, we required at least 2 years of patient‐level data. The first year was used as the base year to identify all index cases of relevant patients that met the specific pre‐specified clinical vignette definition. We then followed patients for 12 months from the index date of hospitalization to measure the service use, costs, and outcomes of the patients. To identify the index cases, we identified all patients in the base year that were hospitalized for the acute event, using a common set of diagnostic codes and relevant procedure codes (Table 1). Across most countries, we used 2 years between 2015 and 2017, except in Australia (2012–2016) and England (2014–2017), which had smaller samples and, therefore, pooled more years of data (Table A3). International classification disease (ICD‐10) codes as defined by the World Health Organization were used from inpatient data files to identify hip fracture patients across all countries. We focused on the codes S72.0, S72.1, and S72.2, which represent fractures of the hip joint. Where ICD‐10 codes were unavailable, such as in Spain (ICD‐9‐CM Codes) and the Netherlands, comparable diagnosis codes were used. Within this group, we then focused on the patients who received one of three procedures: total hip replacement, partial hip replacement, or osteosynthesis—which includes placement of a screw, plate, pin, or internal fixation. Each country used a clinical expert to identify the relevant procedure codes.

**FIGURE 1:**
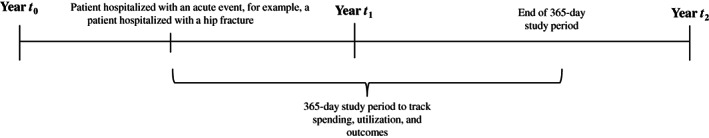
Identification of the high‐need, high‐cost personas across countries. The acute event for the two specific personas included an admission for a hip fracture for the persona with frailty and an admission for a heart failure exacerbation for the persona with complex multimorbidity

For the heart failure persona, we identified all patients hospitalized with a primary diagnosis of congestive heart failure (ICD‐10 code I50.x or relevant codes in Spain and the Netherlands). Given the lack of comprehensive longitudinal data across most countries, we were unable to know if the hospitalization was the first hospitalization related to heart failure or not. We then identified the subset of patients who at the time of the first admission also had a diagnosis of diabetes, including ICD‐10 codes of E11.x, E12.x, E13.x, and E14.x. Once patients were identified, we then tracked from day one of hospitalization all spending, utilization, and relevant patient outcomes that occurred for a period of 365 days (or until date of death if patients did not survive a full year) (Figure 1).

### 2.4 Country selection and datasets

In order to validate our approach, data for the two personas—the older frail adult with hip fracture and the older adult with complex multimorbidity—were extracted from the 11 country databases and examined for comparability. We examined comparability in terms of patient characteristics, including age and sex, and also explored variations in the number of chronic conditions captured in administrative data using Elixhauser definitions.^26^

The participating countries use a range of datasets including administrative claims data, survey data, and registry data. The priority was to have a dataset that captured patient‐level and linked information across different components of the healthcare system. For further details on the representativeness of each dataset and years of data used across countries, see Table A3. The datasets differed with regards to their representativeness of the national population as well as their ability to provide linked data across all seven care settings (Table 2). Further detailed information on the datasets used across the 11 countries is listed in Table A4.

**TABLE 2:** Country dataset information available for public research use in the ICCONIC project

	Australia	Canada	England	France	Germany	Netherlands	New Zealand	Spain	Sweden	Switzerland	United States
Inpatient hospital care	X	X	X	X	X	X	X	X	X	X	X
Post‐acute rehabilitative care		X		X	X	X			X		X
Primary care	X	X	X	X	X	X		X	X		X
Outpatient specialty care	X	X	X	X	X	X	X	X	X	X	X
Home health care		X		X		X		X	X		X
Outpatient drugs	X	X	X	X	X	X	X	X	X		X
Long‐term care		X		X	X	X			X		

Three of the countries identified full national datasets, including New Zealand, Sweden, and Switzerland. New Zealand utilized data from Integrated Data Infrastructure, which is a linked administrative data repository that includes the entire population. Sweden identified data from their national registry and Switzerland identified data made available from the National Health Statistics provided by the Swiss Federal Statistical Office.

Five of the countries identified datasets that captured a large, regionally diverse sample of the population. For example, in the United States, a 20% Medicare fee‐for‐service dataset was identified as one of the data sources. Medicare is the public insurance option available for all people over the age of 65 years and some special groups, including those with disability under the age of 65 and those with end‐stage renal disease. In France, a health insurance claims dataset covering all of the population in 12 regions was used, representing 70% of the French population. Germany used data from the second largest statutory health insurer, BARMER, representing approximately 10% of the German population and active in all regions of the country. England identified the Clinical Practice Research Datalink (CPRD), which is a sample representing around 10% of the England population and links general practitioner's data with hospital records. The Netherlands obtained access to claims data of an insurance company that has a 30% market share across the country.

Three countries identified regional datasets. For example, Australia used data from the Sax Institute's 45 and Up Study, a regionally representative survey of about 10% of the New South Wales population aged over 45, which is the most populous state in the country. In Spain, data from a secure anonymized health information data‐lake (SAHID) covering the entire population of Aragon was used, which represents 3% of the Spanish population. In Canada, data were identified that includes all administrative claims from Ontario, the largest province in the country.

With the identified datasets, three countries—Canada, France, and the Netherlands—had the ability to comprehensively assess care across all seven care domains (Table 2). While Sweden had detailed registry data across all care domains that involve specialized medical doctors, they noted that the primary care data are only accessible at the regional level and had only aggregated and non‐linked data for home health. A further three countries (Spain, Germany, and the United States) were able to analyze the care trajectory across six of the seven domains but not able to examine some of the rehabilitative, home‐health, or long‐term care. Similarly, England was able to identify nationally representative data that would allow for the investigation of four domains, excluding post‐acute rehabilitative care, home‐health, and long‐term care. Switzerland was able to assess relevant patient‐level data in the inpatient setting and hospital‐based outpatient specialty treatment. New Zealand only had access to inpatient, outpatient specialty care, and pharmaceutical data at this time. All 11 countries were able to capture mortality and readmission outcomes specified for the two patient profiles.

### 2.5 Characteristics of the hip persona across countries

Across the 11 countries, sample sizes ranged from *n* = 1859 in Aragon (Spain) to *n* = 29,134 in the United States (Table 3). The mean patient age (standard deviation) ranged from 81.2 years (SD 6.9) in Switzerland to 85.4 years (SD 7.0) in Spain. The sample was predominantly female, with the proportion of women as high as 77.1% in France and the lowest at 62.8% in Australia. Countries varied in the ability to capture secondary diagnoses in the index hospitalization, ranging from an average of 3.7 comorbidities in the United States to 1.1 in New Zealand and Canada. Of note, the Netherlands was unable to calculate the prevalence of secondary diagnoses given that comorbidities could not be captured using the Elixhauser classification in the insurer data.

**TABLE 3:** Sample characteristics of frail older person with a hip fracture with subsequent hip replacement across countries

	Australia	Canada	England	France	Germany	Netherlands	New Zealand	Spain	Sweden	Switzerland	United States
Sample size	2511	9872	2738	42,849	13,998	4463	2940	1859	14,764	6860	29,134
Age											
Mean (SD)	84.7 (7.7)	83.4 (8.1)	83.5 (7.9)	84.3 (7.7)	83.5 (7.7)	82.2 (8.0)	84.0 (7.8)	85.4 (7.0)	83.2 (7.6)	81.2 (6.9)	83.2 (8.3)
Median	86	85	84	85	84	83	85	84	84	82	84
Female (%)	62.8%	70.7%	71.0%	77.1%	75.9%	70.8%	70.4%	76.7%	67.6%	73.7%	71.4%
Chronic conditions											
Mean (SD)	2.9 (1.9)	1.1 (1.2)	2.2 (1.5)	1.7 (1.5)	3.2 (2.1)	n/a	1.1 (1.2)	3.1 (1.5)	2.0 (1.1)	2.1 (1.8)	3.7 (2.0)
Median	2	1	2	1	3		1	3	2	2	3
Diabetes	17.6%	19.5%	15.1%	12.4%	19.8%	n/a	15.0%	20.2%	14.2%	14.2%	22.5%
Heart failure	12.4%	6.0%	10.9%	15.3%	21.6%	n/a	4.7%	8.1%	11.0%	8.4%	17.30%
Depression	3.9%	1.5%	6.9%	5.8%	11.0%	n/a	0.2%	8.3%	2.0%	8.4%	15.3%
Hypertension	24.9%	25.9%	55.0%	41.1%	71.3%	n/a	11.6%	58.5%	41.3%	51.6%	77.1%
Renal failure	12.2%	4.0%	15.2%	6.3%	26.9%	n/a	8.3%	9.0%	5.0%	19.4%	19.2%
Chronic obstructive pulmonary disease	7.3%	6.3%	22.0%	5.0%	10.1%	n/a	4.0%	8.0%	9.5%	8.1%	22.2%
S72.0	1411	4953	1865	26,657	6926	n/a	1665	789	7908	3324	14,548
Total replacement	243 (17.2%)	1138 (23.0%)	220 (11.8%)	5988 (22.5%)	1558 (22.5%)	n/a	297 (17.8%)	56 (7.1%)	1414 (17.9%)	1001 (30.1%)	1492 (10.3%)
Partial replacement	704 (49.9%)	2860 (57.7%)	1184 (63.5%)	12,326 (46.2%)	4424 (62.9%)	n/a	879 (52.8%)	626 (79.3%)	3832 (48.5%)	1934 (58.2%)	9245 (63.6%)
Pinning	464 (32.9%)	955 (19.3%)	461 (24.7%)	8343 (31.3%)	944 (13.6%)	n/a	489 (29.4%)	107 (13.6%)	2662 (33.7%)	389 (11.7%)	3811 (26.2%)
S72.1	973	4381	762	13,543	6045	n/a	1125	843	5710	3147	13,274
Total replacement	14 (1.4%)	95 (2.2%)	Suppressed	396 (2.9%)	88 (1.5%)	n/a	15 (1.3%)	0 (0.0%)	33 (0.6%)	64 (2.0%)	114 (0.9%)
Partial replacement	14 (1.4%)	97 (2.2%)	Suppressed	213 (1.6%)	86 (1.4%)	n/a	12 (1.1%)	10 (1.2%)	31 (0.5%)	68 (2.2%)	317 (2.4%)
Pinning	945 (97.1%)	4189 (95.6%)	706 (92.7%)	12,934 (95.5%)	5871 (97.1%)	n/a	1098 (97.6%)	833 (98.8%)	5646 (98.9%)	3015 (95.8%)	12,843 (96.8%)
S72.2	127	538	111	2649	1027	n/a	150	227	1146	389	1312
Total replacement	Suppressed	13 (2.4%)	Suppressed	66 (2.5%)	15 (1.5%)	n/a	Suppressed	0 (0.0%)	11 (1.0%)	6 (1.5%)	13 (1.0%)
Partial replacement	Suppressed	14 (2.6%)	Suppressed	29 (1.1%)	4 (0.4%)	n/a	Suppressed	14 (6.2%)	7 (0.6%)	20 (5.1%)	26 (2.0%)
Pinning	125 (98.4%)	511 (95.0%)	108 (97.3%)	2554 (96.4%)	1008 (98.2%)	n/a	147 (98.0%)	213 (93.8%)	1128 (98.4%)	363 (93.3%)	1273 (97.0%)

*Note*: The Netherlands was unable to identify Elixhauser conditions or the specific diagnostic codes for hip in the data provided by the insurer. Clinical experts were used to identify the relevant codes in the insurer data that matched the primary diagnoses of interest.

**FIGURE A1:**
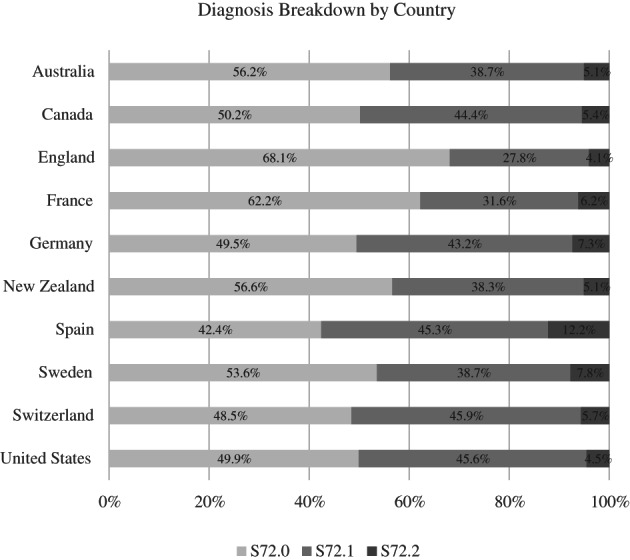
Breakdown of hip fracture diagnoses by country. The data used by the Netherlands did not allow for specific breakdown of individual ICD codes. Clinical experts were used to identify relevant codes for fractures of the upper femur. In Spain, there was a cross‐walk down from ICD‐9 codes to ICD‐10 codes with clinical expert input from the country

**FIGURE A2:**
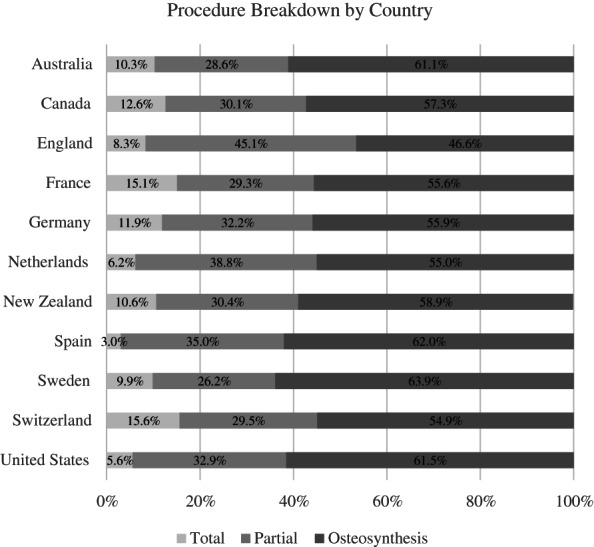
Breakdown of type of procedure by country. Total, total hip replacement; Partial, partial hip replacement

In all countries, except in Spain, the most common diagnostic code was S72.0: ranging from 68.1% of the sample in England to 42.4% of the sample in Switzerland. This was followed by S72.1 and then S72.2 (see Figure A1). Within the different diagnostic codes, the breakdown of procedure type differed. Among patients with the S72.0 code, the most common procedure across countries was a partial hip replacement (see Figure A2). For the patients with S72.1 and S72.2, the most common procedure was osteosynthesis (e.g., internal fixation using screws, screw‐rod, or screw‐plate constructs). These findings are aligned with the sociodemographic, clinical, and treatment‐based characteristics of recent studies evaluating hip fracture patients in large‐scale datasets.^27^, ^28^, ^29^

### 2.6 Characteristics of the hospitalized heart failure persona with diabetes across countries

All countries were able to identify the heart failure persona with diabetes as a comorbidity. England had the lowest sample size (*n* = 742), whereas the United States (*n* = 21,803) and France (*n* = 21,957) had the largest sample sizes (Table 4). The mean age (standard deviation) ranged from 76.2 (SD 5.6) years in the Netherlands to 80.3 (SD 6.8) years in Sweden. The proportion of female patients ranged from 36.5% in Australia to 50.0% in the United States.

**TABLE 4:** Sample characteristics of an older person hospitalized with heart failure with a comorbidity of diabetes

	Australia	Canada	England	France	Germany	Netherlands	New Zealand	Spain	Sweden	Switzerland	United States
Sample size	3014	6305	742	21,957	10,583	2035	1572	1270	4615	3369	21,803
Age											
Mean (SD)	79.4 (6.8)	78.1 (7.0)	78.7 (6.6)	79.1 (6.9)	79.0 (6.4)	76.2 (5.6)	77.3 (6.9)	80.2 (5.1)	80.3 (6.8)	78.6 (6.5)	77.2 (7.0)
Median	80	79	79	80	79	77	77	81	81	77	77
Proportion female	36.5%	45.4%	42.5%	46.0%	50.7%	48.4%	42.4%	46.9%	41.3%	42.6%	50.0%
Chronic conditions											
Mean (SD)	5.9 (2.3)	3.5 (1.3)	5.1 (1.5)	6.8 (2.5)	6.1 (2.0)	n/a	3.9 (1.4)	5.6 (2.1)	3.2 (1.2)	5.9 (1.7)	6.3 (1.7)
Median	6	3	5	5	6	n/a	4	5	3	6	6
Chronic conditions											
Heart failure	100%	100%	100%	100%	100%	100%	100%	100%	100%	100%	100%
Diabetes	100%	100%	100%	100%	100%	100%	100%	100%	100%	100%	100%
Depression	4.0%	1.5%	3.5%	4.7%	6.3%	n/a	0.4%	6.6%	1.0%	8.0%	1.5%
Hypertension	51.6%	41.1%	64.2%	69.0%	82.4%	n/a	51.2%	75.7%	57.2%	81.2%	89.3%
Renal failure	49.3%	5.5%	39.2%	34.9%	59.0%	n/a	47.3%	42.9%	25.8%	62.1%	54.5%
Chronic obstructive pulmonary disease	26.2%	16.3%	31.0%	17.1%	22.7%	n/a	10.3%	24.6%	18.7%	17.7%	42.7%

*Note*: The Netherlands was unable to identify Elixhauser conditions in the data provided by the insurer. Clinical experts were used to identify the relevant codes in the insurer data that matched the primary diagnoses of interest.

Countries captured a different number of comorbidities among their sample. Canada recorded the fewest number of chronic conditions with a mean (SD) of 3.5 (SD 1.3), whereas Germany and the United States had the highest with a mean (SD) of 6.1 (SD 2.0) and 6.3 (SD 1.7), respectively. Of note, the Netherlands was unable to calculate the prevalence of secondary diagnoses given that comorbidities could not be captured using the Elixhauser classification in their insurer data.

### 2.7 Ability to track utilization, spending, and outcomes across different care domains

All countries were able to identify individuals corresponding to both personas, but not all of them could track these individuals across seven domains of care with the data available. The seven domains of care included (1) hospital inpatient care, (2) post‐acute rehabilitative care, (3) primary care, (4) outpatient specialty care, (5) outpatient pharmaceuticals, (6) home‐health care, and (7) long‐term care.

While all countries are able to report data on expenditures, there are differences in what information they are able to report on the different care settings (Table A1). In addition, the cost accounting methods used to estimate expenditure differ across countries, in part due to the differences in payment systems adopted, which also vary across care settings within countries. For example, some countries are able to report direct spending from incurred costs (those with full costing systems), whereas others provide information on reimbursement for specific episodes (e.g., diagnosis‐related groups) or an unweighted average unit price. In addition, the reporting and imputation of capital investments or indirect costs also varies across system.

## 3 DISCUSSION

In this study, we present a novel methodological framework for identifying two specific types of HNHC patient personas that reflect a priority population for most healthcare systems. The two personas are an older adult with frailty, personified by an individual age 65 and older who sustained a hip fracture, and an older adult with complex multimorbidity, including heart failure and diabetes. Our work highlights a framework that allows for international comparisons across countries using routinely collected data linked across different care settings.

This article serves as the methodological supplement for a series of six additional papers copublished in the *Health Services Research* “Special Issue on International Comparisons of High‐Need, High‐Cost Patients.” The first two papers examine cross‐country variations in healthcare utilization and spending for the two personas: the older person with complex multimorbidity (including heart failure and diabetes),^11^ and the older adult with frailty that experienced a hip fracture with subsequent hip replacement.^12^ These papers present a series of comparative metrics collated by the research collaborative, which examine the relative resource use made across care settings by these patients over the course of their care. For the hip fracture persona, this resource use is compared to the cohort's utilization and spending in the year prior to their hospitalization. For the multimorbid persona, the resource use is compared to patients with fewer or additional comorbidities. The third paper then presents a comparison of patient outcomes across these two personas, including hospital readmissions and patient mortality.^9^

In order to better understand how resource use may vary for those who die during the course of the study period, a separate paper examines spending and utilization at the end of life among people with hip fracture.^13^ A fifth paper examines the within‐country variation of healthcare utilization and spending among people with complex multimorbidity (heart failure and diabetes) to better understand if there are important inequalities in care utilization across countries.^14^ Finally, for countries that have access to data on long‐term care utilization and spending, an accompanying sixth paper examines the relative use of different types of long‐term and post‐acute care use among those who experience a hip fracture.^10^

A key strength of the methodology presented in this article is that it segments the HNHC population into distinct patient types who require different types of care from the health system and therefore move across the care settings differently. We identified the two chosen personas from a hospitalization as the initial inciting event. All 11 countries in our study had reliable hospital data that allowed the identification of the hip fracture persona and the complex multimorbid persona with heart failure and diabetes.

Furthermore, the use of the HNHC patient persona approach offers some standardization across participating countries that is important for two reasons. First, most existing comparative studies of high‐cost patients to date have focused on comparing a broad group of patients that make up the top 1%–10% of health care spending in different countries. The types of patients found in this group are quite heterogenous and therefore make it challenging for these comparisons to yield actionable insights for policymakers.^1^, ^30^ In contrast, our approach is designed to identify homogenous groups of HNHC personas that allow for more comparability across countries.

Second, the merit of applying a clinical vignette approach is that by applying specific diagnostic and age criteria to identify patients across countries, we are able to compare a more similar cohort of individuals. This is necessary given the many factors that can influence patient resource use and outcomes across countries, such as structural differences in how they capture and incentivize coding of primary and secondary comorbidities, which limits the ability to do robust risk adjustment. For example, for inpatient conditions, many countries utilize diagnosis‐related groups. Other countries have financial incentives to code for certain conditions, like diabetes in England and dementia in France.^31^, ^32^ Therefore, in our identification of priority populations, we prioritized complex diagnoses that are often coded across all settings given that they require substantial attention.

There are important limitations to our approach that are relevant for all six accompanying papers copublished in the HSR special issue. First, limitations in data type, structure, and availability may influence our identification strategy of the personas across countries. For example, not all countries use ICD‐10 codes, and even ICD‐10 codes may differ by country and year. In addition, the Netherlands required clinical data from the private insurer to be transcribed by clinical experts to match the ICD codes, and therefore, it is possible that there may be differences in which patients get selected into the sample. The use of the patient vignette that is dependent on hospital data, however, may help us overcome these barriers given that it likely increases the specificity of the persona. Second, there are important differences in national costing and coding practices between countries. For example, countries such as Germany and the United States have strong incentives to upcode secondary comorbidities in the index hospitalization since it may affect their payment amount, which is not as important in other countries, such as Canada, that recorded the fewest number of comorbidities. Therefore, differences in comorbidities likely reflect structural differences rather than severity of illness, which may limit our ability to risk‐adjust using comorbidities.

There are also important limitations in the ability to identify other types of HNHC personas. For example, the NAM identified other priority populations, including those with serious mental illness and older adults with dementia. However, the identification of personas that require the use of diagnostic codes in primary care or outpatient specialty care settings is not possible in many countries. Only five countries have the ability to capture chronic conditions in non‐hospital settings. We also observe large differences in data availability, which may influence generalizability within countries. In some countries, regional samples were used (e.g., Canada, Spain, and New Zealand), which may not be representative for other populations in the country. In addition, many countries have gaps in their ability to follow patients across the entire care pathway. For example, data on long‐term care was lacking across most countries. Linked post‐acute care and home care was also not available for research use in many countries.

## 4 CONCLUSION

Our study evaluated the feasibility of utilizing a novel international comparisons approach that examines the complete care trajectory of HNHC patients. Although there are cross‐country differences in the availability and structure of data sources across countries, it is reassuring that all 11 countries had the ability to effectively participate in this approach. This article serves as the methodological supplement to an accompanying six additional papers that further evaluate differences in spending, utilization, and patient outcomes for the two personas. It also provides a blueprint for other countries who may be interested in performing international comparisons of HNHC populations. Such developments can yield important insights on how best to deliver care for complex patients and improve allocation of resources in countries that share similar populations and problems.

## Appendix

**TABLE A1:** Health system characteristics by country

Health system characteristics	Australia	Canada	England	France	Germany	Netherlands	New Zealand	Spain	Sweden	Switzerland	United States
Health system expenditure per capita (2019)	$5187	$5418	$4653	$5376	$6646	$5765	$4204	$3616	$5782	$7732	$11,072
Long‐term care expenditure per capita (2017)	$101	$923	$726	$780	$1099	$1385	—	$317	$1425	$1409	$512
Health system expenditure % of GDP (2019)	9.3%	10.8%	10.3%	11.2%	11.7%	10.0%	9.3%	9.0%	10.9%	12.1%	17.0%
Long‐term care expenditure % of GDP (2017)	0.2%	1.9%	1.8%	1.8%	2.1%	2.6%	—	0.9%	2.9%	2.4%	0.9%
Type of system (NHS type, private insurance, social insurance)	National public health insurance	National public insurance	National healthcare system (NHS)	Statutory insurance through employment‐based funds, tax‐financed coverage for unemployed	Mostly statutory insurance with some private insurance	Statutory, mandatory insurance through 11 private nonprofit carriers	National healthcare system	National healthcare system	National healthcare system with decentralized service delivery	National health insurance for basic coverage with optional supplementary insurance plans	Mix of public and private insurance
Population coverage (%)	100%	100%	100%	100%	100%	100%	100%	100%	100%	100%	91.5%
Payment system of hospitals	Public hospitals mostly activity‐based (DRG) payments, with the rest global budgets while private hospitals mainly FFS	Global budgets, some case‐based payment	Mostly case‐based payments, with some local bundled‐payment pilots	Mostly case‐based (DRG) payments	Case‐based (DRG) payments	Case‐based (DRG) payments with a global budget	Case‐based payments	Mostly global budgets, some episode‐based payments	Mostly global budgets, remainder case‐based (DRG) payments or PFP	Case‐based (DRG) payments for inpatient care, FFS for outpatient care	Mix of FFS, case‐based (DRG), and per‐diem payments
Payment system of primary care (FFS, capitation, PFP, hybrid)	Mostly FFS, some PFP	Mostly FFS, some alternative payments or salaries	Mix of capitation, FFS, PFP	Mix of FFS and PFP, capitated annual bonus for chronic diseases	FFS	Mix of capitation and FFS, some bundled payments and PFP	Capitation and FFS, some incentive payments	Global budgets, capitation, PFP	Mostly capitation, some FFS or PFP	Mostly FFS, some capitation	Mostly FFS, some capitation and incentive payments

Abbreviations: DRG, diagnosis‐related group; FFS, fee‐for‐service; GDP, gross domestic product; NHS, National Health Service; PFP, pay for performance.

**TABLE A2:** ICCONIC advisory board members

Country	Member	Title
Meeting Chairs	Peter C. Smith, MSc	Emeritus Professor of Health Policy, Imperial College London
	Melinda K. Abrams, MS	Senior Vice President, Delivery System Reform and International Innovations, The Commonwealth Fund
	Andrew Street	Professor of Health Economics, London School of Economics and Political Science
Australia	Philip Haywood	Senior Research Fellow, Centre for Health Economics Research and Evaluation
	Sallie Pearson	Professor, UNSW Centre for Big Data in Health Research
	Jason Thompson	Unit Head | Economics, Expenditure & Medicare Unit | Australian Institute of Health and Welfare
Canada	Rhona McGlasson, MBA, PT	Executive Director, Bone and Joint Canada
	Fredrika Scarth, PhD, MA	Director, Secretariat, Premier's Council on Improving Healthcare and Ending Hallway Medicine, Ontario Ministry of Health
England	Peter C. Smith, MSc	Emeritus Professor of Health Policy, Imperial College London
	Antony Johansen, MBBS	Consultant Orthogeriatrician, University Hospital of Wales Clinical Lead, National Hip Fracture Database, Royal College of Physicians
France	Sandrine Colas, PhD	Pharmacoepidemiologist, Real World Insights
	Antoine Rachas, MD, PhD	Public Health Doctor, French National Health Insurance (CNAM)
Germany	Reinhard Busse, Dr Med, MPH	Professor and Head of the Department of Health Care Management, Berlin University of Technology
	Jens Deerberg‐Wittram, MD	CEO, RoMed Kliniken
Netherlands	Patrick Jeurissen, PhD, MPA	Professor, Radboud University Medical School Science Officer, Ministry of Health, Welfare and Sports
New Zealand	Richard Hamblin	Director for Health Quality Intelligence at our Health Quality and Safety Commission
	Lisa Gestro	Executive Director of Primary and Community Strategy, Southern District Health Board
Spain	Ismael Said, MD, MSc	Specialist in Internal Medicine, Hospital Universitario Ramón y Cajal
	Francisco Estupiñán‐Romero, MD	Researcher, Health Science Institute in Aragón (IACS)
	Carlos Martín Hernández, MD, PhD, MSc	Specialist, Orthopedic Surgery and Traumatology Associate Professor of Orthopedic Surgery and Traumatology, Zaragoza University
Sweden	Jean‐Luc af Geijerstam, MD, PhD	Executive Director, The Swedish Agency for Health and Care Services Analysis
	Cecilia Rogmark, MD, PhD	Orthopedic Surgeon, Skane University Hospital Associate Professor, Lund University
Switzerland	Lars Clarfeld, Dr Med	General Secretary, Swiss Society of General Internal Medicine (SGAIM)
United States	Eric Schneider, MD MSc	Senior Vice President for Policy and Research, The Commonwealth Fund
	Andrew Schoenfeld, MD, MSc	Orthopedic Surgeon, Brigham and Women's Hospital Associate Professor of Orthopedic Surgery, Harvard Medical School

**TABLE A3:** Representativeness of country datasets

	Australia	Canada	England	France	Germany	Netherlands	New Zealand	Spain	Sweden	Switzerland	United States
Year of data	2012–2016	2016–2017	2014–2017	2016–2017	2016–2017	2016–2017	2016–2017	2015–2016	2015–2016	2015–2016	2016–2017
Country population											
Total	23.5 million	35.2 million (Canada 2016)	55.3 million (in 2016)	64.5 million	82.2 million	16.9 million	4.7 million	46.6 million	9.9 million	8.4 million	323.1 million
Population age 65+	3.5 million	5.9 million (Canada 2016)	9.9 million (in 2016)	12.3 million	16.8 million	2.4 million	733,272	8.3 million	1.9 million	1.5 million	49.2 million
Sample population (dataset)											
Representativeness of dataset	Regional data (New South Wales)	Regional data (Ontario Province)	National sample	12 regions of France	National sample	National sample	Full national data	Regional data (Aragon, Spain)	Full national data	Full national data	National sample
Total sample in dataset	267,086	14.5 million	7% of the UK population, representative in terms of age and sex	43.1 million	8.6 million	30% market share	4.7 million	1.3 million	9.9 million	8.4 million	6.4 million
Population age 65+ in dataset	123,625	2.5 million		8.2 million	2.5 million		733,272	288,738 (21.73%)	1.9 million	1.5 million	5.4 million

*Note*: Specific full denominator was not able to be shared by data supplier in England and in the Netherlands. In Australia, the full sample of the 45 and up study cohort at baseline is *n* = 267,153 but for this research sample was 267,086.

**TABLE A4:** Country datasets used for comparison

Country	Datasets
Australia	Sax Institute's 45 and up study (see note)
Canada	Administrative claims data of the province of Ontario from the Ontario Ministry of Health and the Canadian Institute for Health Information through the Institute for Clinical Evaluative Sciences
England	Primary care data from the Clinical Practice Research Datalink linked to secondary care data from Hospital Episode Statistics and Office for National Statistics death register
France	SNDS (Système National des Données de Santé/National Health Data System) ResidEhpad (long‐term care in residential facilities)
Germany	Administrative data of a large, nationally active health insurance with more than 8 m enrollees (BARMER) (includes utilization/costs of all sectors that are paid by health insurance)
Netherlands	Zilveren Kruis insurance data (nationwide), which has about 30% of market share in the country
New Zealand	The Integrated Data Infrastructure The National Minimum Dataset (hospital admissions data) The pharmaceutical collection (medication dispensing data) The National Non‐Admitted Patient Collection (outpatient data)
Spain	Base de datos de usuario (National Health Service users dataset including insurees admin data) OMI‐AP (primary care electronic health records) Conjunto Mínimo Básico de Datos (administrative data for hospital discharges and outpatient contacts) Sistema de Información Hospitalaria (outpatient visits to specialized care) Receta Electrónica (e‐Prescription files) Facturación Recetas (billing files of over‐the‐counter prescriptions) Puesto Clínico Hospitalario de Urgencias (emergency care contacts)
Sweden	The national patient registry (inpatient and outpatient specialized care) The national prescription drug registry (outpatient pharmaceuticals) The national mortality registry The national registry for interventions in municipal health care (enrollment in home medical care) The national registry of measures for the elderly and people with disabilities (long‐term care) Regional administrative registers of primary care consumption for the regions of Stockholm, Jönköping, Norrbotten, Skåne, and Västra Götaland.
Switzerland	Medical statistics dataset of the Federal Statistical Office (FSO), including hospital admissions records Patient data from hospital‐based outpatient care dataset of the FSO Short‐ and long‐term care facility records dataset of the FSO
United States	Medicare fee‐for‐service data, 20% sample of all patients age 65 years or older

*Note*: This research representing Australia was completed using data collected through the 45 and Up Study (www.saxinstitute.org.au)—a sample of people above the age of 45 in New South Wales (NSW), Australia.^33^ The 45 and Up Study is managed by the Sax Institute in collaboration with major partner Cancer Council NSW; and partners: Heart Foundation; NSW Ministry of Health; NSW Department of Communities and Justice; and Australian Red Cross Lifeblood. We thank the many thousands of people participating in the 45 and Up Study. We also acknowledge Services Australia (formerly the Australian Government Department of Human Services) for the provision of Medicare Benefits Schedule (MBS) and the Pharmaceutical Benefits Scheme (PBS) datasets. The Admitted Patient Data Collection (APDC) and Emergency Department Data Collection (EDDC) data were provided by the NSW Ministry of Health and linked to the 45 and Up Study data by the Centre for Health Record Linkage (CHeReL, see http://www.cherel.org.au/ for more details). We capture the administrative health datasets of each survey participant by linking the survey to each component. Linkage to APDC and EDDC is undertaken by CHeReL using probabilistic matching, whereas linkage to MBS and PBS is undertaken by the Sax Institute using the deterministic matching method. All datasets are accessed within the Secured Unified Research Environment (SURE) provided by the Sax Institute. The 45 and Up Study has ethical approval from the University of New South Wales (UNSW) Human Research Ethics Committee. This study has received ethics approval from the UTS Human Research Ethics Committee (UTS HREC REF NO. ETH18‐2507) and the NSW Population & Health Services Research Ethics Committee under reference number 2013/11/487. The Sax Institute's 45 and Up Study survey data used to represent Australia oversamples people above age 80 and residents of rural and remote areas.^33^ The 45 and Up Study had a response rate of about 18%, so the cohort might not be representative of the NSW population. Also, the survey focuses on NSW and may not be representative of the national sample for the same age group.
